# Individually designed fall prevention strategies compared to generic strategies for mastering complex fall risk situations in people with multiple sclerosis: study protocol of a randomised controlled trial

**DOI:** 10.1136/bmjopen-2025-115430

**Published:** 2026-07-15

**Authors:** Ylva Nilsagård, Ruzan Udumyan, Maria Halleberg Nyman, Anna Wäneskog

**Affiliations:** 1 University Health Care Research Center, Örebro University Faculty of Medicine and Health, Örebro, Sweden; 2 Clinical Epidemiology and Biostatistics, Örebro University Faculty of Medicine and Health, Örebro, Örebro County, Sweden; 3 Faculty of Medicine and Health, University Healthcare Research Center, Örebro, Örebro County, Sweden; 4 Center for Innovation, Research and Education, Västerås Central Hospital, Västerås, Västmanland County, Sweden

**Keywords:** falls, intervention, multiple sclerosis, physiotherapy, prevention

## Abstract

**Introduction:**

Of individuals with mild-to-moderate multiple sclerosis (MS), 56% report falling at least once during a 3-month period. Several fall risk factors have been identified, but the issue is complex, with interactions between triggering factors and preceding activities and events. There is a lack of studies explicitly evaluating fall prevention strategies given as counselling over time.

**Methods and analysis:**

The project includes (1) a two-armed randomised controlled internal pilot study with a nested qualitative study on participants’ experiences and (2) a randomised controlled trial (RCT). The pilot study will evaluate feasibility in terms of recruitment, dropout, adverse events and battery of tests and will constitute a basis for recalculating the preliminary estimated sample size for a full-scale study. The RCT study will evaluate whether fall prevention strategies based on individual fall risk evaluation reduce fall frequency compared with general fall prevention information. Participants in the intervention group will have an extensive discussion with a physiotherapist regarding the impact of specific MS symptoms, environmental and personal factors, triggering factors and activities and/or circumstances that they perceive to precede fall situations; these discussions will then lead to the creation of individual strategies. In case of falling during follow-up, further discussions on strategies will be held by phone contact. The control group will receive general fall risk prevention recommendations. After completion of the study, the control group will be offered individual strategies based on reported falls. Participants in the pilot study allocated to the intervention group will after follow-up be invited to individual interviews focusing on experiences of taking part in the intervention and the study.

Adults diagnosed with MS, fall history and remaining walking ability will be recruited by physiotherapists from six sites in Sweden. The primary outcome will be self-reported falls for 6 months and secondary outcomes will be self-rating scales covering concern about falling, confidence in remaining balance during activities, walking limitations and ability to avoid falls. Descriptive measures of disease impact will be used.

**Ethics and dissemination:**

The study was approved by the Swedish Ethical Review Authority, Stockholm Dept. 4 (ID: 2025-04486-1, date: 2025-08-12). All participants will provide written informed consent. Findings will be disseminated through peer-reviewed journals, conference presentations and relevant patient organisations.

**Trial registration:**

NCT07378566.

**STRENGTHS AND LIMITATIONS**

- Using both quantitative and qualitative methods to inform this study will enable a multidimensional and in-depth exploration.
- A patient representative has been a partner throughout the designing and planning of the study.
- The internal pilot study can be integrated in the randomised controlled trial.
- The multi-centre design with many different deliverers of the intervention may increase the generalisability but also decrease fidelity.
- Self-reported outcome data can be a potential limitation.

## Introduction

 Approximately 2.8 million people around the world have multiple sclerosis (MS); the mean age of diagnosis is 32 years.^1^ Balance impairments are reported at an early stage of MS,^2 3^ and disease progression increases the risk of falls. People with MS have a higher risk of falling than even the general population of older people^4^; 56% of those with mild-to-moderate MS reported falling at least once during a 3-month period,^5^ and 60% express fear of falling.^6^ The fall risk peaks when limitations in walking ability first begin, and when there is a need to start using a walking device.^5^ Several fall risk factors have been identified, such as medicines, history of falling,^7^ divided attention, fatigue and reduced muscular endurance,^8^ low confidence in the ability to avoid falling^9^ and variations within and between days.^10 11^ Other factors are environmental, such as slippery or uneven walking surfaces, use of a walking device and crowded places.^8 10 12^ The complexity of falls, including the interaction of triggering factors and preceding activities and events, has been described in a recent qualitative study.^13^

Falls may result in several negative consequences such as injuries,^14^ avoidance of being active and reduced quality of life.^9^ Fear of falling, or concerns about falling, may lead to a negative spiral where activities are avoided.^15^ Taken together, this may negatively impact participation in society, for example, in terms of working life and leisure activities. It is therefore of the utmost importance that physiotherapists have knowledge not only of fall risk factors but also the interaction between those factors, to be able to provide effective interventions that strengthen the individual’s ability to find effective fall-reducing strategies.

There is a large inter-individual variation of level of functioning and level of activity in people with MS, and so it is essential to tailor and evaluate interventions that can be adapted for an individual person and their specific situation. In the present study, fall prevention strategies will be designed regarding the resources and circumstances of each individual. This is important because falls most commonly occur in everyday situations, which are difficult to avoid.^10 13^ Since fatigue is common in people with MS, interventions using remote solutions may save energy, reducing the travel time and effort needed to attend care visits. Remote solutions, for example, using short message service and phone calls as in the present study, can thereby increase the accessibility of an intervention.

Rehabilitation professionals often use combinations of supervised and non-supervised exercise and advice to achieve the desired effect. There has been some criticism of combining interventions, as these act as a so-called ‘black box’ and make it difficult to distinguish which components are effective and which are not. The present study responds to that criticism by explicitly studying the effect and experiences of individual counselling for fall prevention strategies among people with MS, bridging over the problem of evaluating interventions that comprise several components. It can provide unique knowledge about the effect and experiences of individual fall prevention strategies that consider environmental factors, personal factors and the activities and circumstances preceding falls, alongside MS symptoms. This intervention may strengthen the individual’s ability to manage fall risk situations and reduce concerns about falling, which may increase their ability to be physically and socially active. The study starts with an internal pilot study with a nested qualitative study to inform a randomised controlled trial (RCT). If the results are promising, the intervention can be included in a multi-strategy’ intervention.

## Methods and analysis

The present study protocol is reported according to the Standard Protocol Items: Recommendations for Interventional Trials (SPIRIT) guidelines.^16^ *The* RCT aims to evaluate whether a fall prevention strategy based on individual fall risk evaluation will reduce fall frequency in people with MS compared with general fall prevention information. The hypothesis is that the intervention group will report 30% less falls during the 6-month period when the intervention is active, compared with the control group. This was based on clinical reasoning, considering that healthy people also fall,^17^ that people with MS often fall during everyday activities that are difficult to avoid,^5 10^ that behavioural change takes time^18^ and that the control group receives fall-related information that may influence fall outcomes.

*The internal pilot study* will evaluate feasibility in terms of recruitment, dropout, adverse events and battery of tests and will constitute a basis for recalculation of the preliminary estimated sample size. Internal pilot studies allow for adjustments while still allowing all participants to be included in the RCT.^19^

*The qualitative study* nested in the pilot-study will describe participants’ experiences of the intervention, as well as how they experience the data collection methods. Adding a qualitative perspective provides an opportunity to gain a deeper and more nuanced understanding regarding how the intervention may contribute to mastering of fall risk situations.

### Design

A parallel group design with random 1:1 allocation to control or intervention group will be used for the internal pilot study and the RCT. The internal pilot will include centres identified for the main trial to ensure a more realistic assessment of feasibility (recruitment, retention, data collection, adherence) and may provide empirical estimates to refine sample size assumptions for the main trial. If no major protocol changes are required, pilot data will be retained for the final analysis, thereby improving overall study efficiency.

To minimise risks of bias and operational inconsistency, progression criteria will be pre-specified, any sample size re-estimation will follow predefined methods, and protocol modifications will be kept to a minimum and fully documented, ensuring consistency between the pilot and main phases and preserving study validity.^20^ The findings will be reported according to the Consolidated Standards of Reporting Trials (CONSORT) statement extension for randomised pilot and feasibility trials.^21^ Due to the nature of the intervention, neither the participants nor the physiotherapists providing the intervention will be blinded. However, the statistician conducting the analyses will be blinded to group allocation. The qualitative study will have an explorative and descriptive design^22^ and will be reported in adherence with the Consolidated Criteria for Reporting Qualitative Research (COREQ) checklist.^23^

### Patient and public involvement statement

A patient representative with personal experience of falling, recruited by a MS team nurse in Region Örebro County, contributed to the study planning. Patient involvement included discussions about the overall relevance of the project and its specific objectives. Points of view were collected regarding the extent and process of the intervention, the control condition, and the data collection. The time and effort required to participate in the study, including completing the questionnaires and taking part in the follow-ups, were discussed and found to be reasonable.

Patient involvement also included points of view on the readability and comprehensibility of the study information. Potential risks and benefits were discussed and considered in the ethics application. The collaboration was formalised according to the current guidelines of Region Örebro County, which include reimbursement to patient representatives. The patient representative was not involved in the recruitment or conduct of the study. Both parties are open to further collaboration regarding the presentation and dissemination of the results, but this has not yet been formalised.

### Setting

Physiotherapists (two per centre) trained in neurological physiotherapy will include participants at six centres in Sweden: Department of Neurology and Rehabilitation Medicine, University Hospital, Örebro; Department of Physiotherapy, Hudiksvall Hospital, Hudiksvall; NeuroRehab Unit, Mälar Hospital, Eskilstuna; Department of Physiotherapy, Gävle Hospital, Gävle; Neurological Rehabilitation Unit, Västerås Hospital, Västerås; and NeuroRehab, Nyköping Hospital, Nyköping. Potential participants will be identified through contact with the relevant department.

### Characteristics of participants

The inclusion criteria will be aged 18 years or older, having a diagnosis of MS, still being able to walk but having a self-assessed decline in the ability to walk due to MS and at least one self-reported fall due to MS during the last 3 months.

The exclusion criteria are judged by the respective physiotherapist and will be: any difficulty filling in self-rating scales, being unable to walk 100 m (walking aid and intermittent rest allowed) and having a balance impairment clearly related to other neurological or orthopaedic diagnoses.

### Intervention

Individual fall preventative strategies, based on a fall risk analysis supported by the International Classification of Functioning, Disability and Health (ICF),^24^ will be added to general fall risk information. The ICF constitutes two domains with two components per domain: functioning and disability (body function/structure and activities/participation) and contextual factors (environmental and personal). During a visit to the physiotherapist clinic, the individual will be asked to describe recent fall situations. The physiotherapist and the participant will discuss the impact of specific MS symptoms, environmental and personal factors, triggering factors and activities and/or circumstances that the individual perceives to precede fall situations, see table 1 for examples. The discussion will lead to jointly agreed fall preventative strategies.

**Table 1:** Examples of follow-up questions and things to discuss to identify individual strategies to reduce fall risk

Two-and three level ICF categories	Example of follow-up questions	Example of things to discuss—strategies
Energy and drive functions b130; energy level b 1300	Identification of patterns over the day, doing what, preceding activities, under what circumstances, physical or mental fatigue	Find pauses, physical activity, plan activities, respect your limits, make active choices, ask for help
Sustaining attention b1400; dividing attention b1404	During specific activities/circumstances Relation to stress, fatigue, environment	Isolate activities, reduce environmental stress, plan activities
Sensitivity to temperature b2700	During physical activity, indoor or outdoor temperature	Cooling garment, cooling regimes, devices.
Gait pattern functions b770	What happens, where and when? (visual inspection)	Shoes, furnishing, surfaces, choose environment/activities, use of walking device, orthotic device
Lifting and carrying objects d430	In what situations, carrying what, moving how far, in what environment, how heavy/large?	Carry in a bag instead of in the hands, use bags with large handles or a backpack, use items with rough surface
Balance b235 (in relation to body functions)	In what situations, what happens and when?	Broaden the support base, find lower level (sitting instead of standing), find external support

ICF, International Classification of Functioning, Disability and Health.

Using the time when the participant is filling in the self-report questionnaires, the physiotherapist will document the agreed individual fall prevention strategies and will then go through it with the participant at the end of the visit. A teach-back method is used, in which participants repeat the strategies back to the physiotherapist to ensure mutual agreement and to reinforce the strategies. Participants are encouraged to share the strategies with at least one next-of-kin after the visit and to place the written strategies somewhere at home where they will be seen daily. We estimate the duration of the physical visit to be approximately 90 min and will monitor this estimate during the internal pilot study. If the participant reports falling during the following 6-month period, a reinforced strategy will be formulated including the information from the reported fall(s). Participants allocated to the intervention group will be invited (within 2 weeks after the end of the intervention) to take part in the qualitative study.

### Control

**Figure 1:**
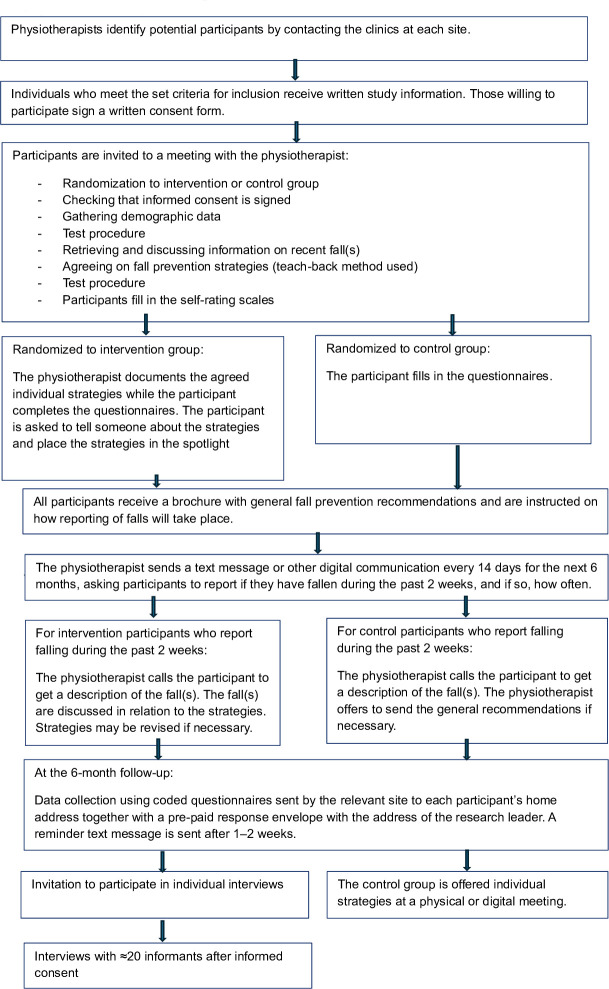
Study protocol flow chart

The control group will receive written general fall risk information; this information recommends continuing to be physically active, using the recommended walking device, using shoes that fit their feet well, paying attention to the ground when walking, being aware of how their body reacts to stress and planning for pauses. All individuals in the control group will be offered the study intervention after the study ends at 6 months, based on all their reported falls. A flowchart of the procedures and a participant timeline^25^ are given in figure 1 and table 2.

**Table 2:** Participant timeline: schedule of enrolment, allocation and measurements

	Trial period					
	**Internal pilot and full scale RCT**					**Qualitative study**
	**Enrolment**	**Allocation**	**Baseline**	**Every 2 weeks for 6 months**	**Follow-up**	**Enrolment**
**TIMEPOINT**	*-t1*	*t1*	*t2*	*t3*	*t4*	*t5*
ENROLMENT						
Eligibility screen	x					
Informed consent	x					x
Check for criteria		x				
Randomisation		x				
INTERVENTION/ COMPARATOR						
*Individual fall prevention strategy as add-on to general fall risk information*			x	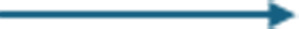 when reporting falls		
*General fall risk information*			x	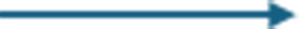 when reporting falls		
ASSESSMENTS						
*Demographic data*			x*		x†	
*Timed Up and Go Test, Six Spot Step Test, Symbol Digit Modalities Test, 29-item MS Impact Scale*			x			
*Falls/no falls, adverse events*				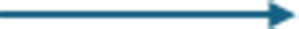		
*Number of falls, description of falls, injury due to fall, need to seek care due to fall*				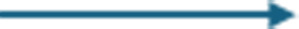 when reporting falls		
*Falls Efficacy Scale International, Activities-Specific Balance Confidence Scale, 12-item MS Walking Scale, Frändin-Grimby Physical Activity Scale, fear of falling, avoiding activities due to fall risk, ability to prevent falls, understanding fall risk factors, self-rated change in balance*			x		x	
*Individual interviews*						x

* Demographic data: sex, gender, MS type, falls during the past 3 months, ongoing physiotherapy/rehabilitation, walking device, occupation, living condition, contact information.

† Exacerbation, ongoing physiotherapy/rehabilitation, changes in walking device use, living condition or occupation.

MS, multiple sclerosis; RCT, randomised controlled trial.

### Outcome measures

#### The internal pilot study

Feasibility of the recruitment process will consider

1. The proportion of people with MS that agree to participate calculated as the ratio of invited and agreed. We expect an agreement rate at ≈50%.
2. The proportion of invited and agreed that met all criteria for inclusion. We expect that less than 15% of those agreeing to participate will fall out due to inclusion or exclusion criteria.
3. Inclusion rate. We expect the estimated number of participants in the pilot study (n=35–40) to be included within the study’s first year.

Feasibility of the data collection procedures will consider

1. If the estimated time for the visit (90 min) was sufficient.
2. The physiotherapists’ clinical judgement of participant effort to fill in the questionnaires and perform the physical and cognitive tests, discussed repeatedly during the study period.
3. The narratives from the informants in the individual interviews.

Feasibility of the outcome measures will consider

1. Completed questionnaires at baseline and follow-up.
2. Preliminary analyses showing trends of between-group differences.

Feasibility of the intervention will consider

1. The physiotherapists’ professional opinion on feasibility.
2. The informants’ narratives on whether they would recommend the intervention (from the interviews).

*Retention rate* will consider proportion of dropouts from baseline to follow-up. We expect a drop-out rate at approximately 10%. The ethical approval does not allow us to ask for reasons for dropout but will be noted if the participant expresses reasons spontaneously.

*Potential harms* are defined as falls for which the participants made a direct connection to failed strategies; that is, strategies that were intended to prevent falling but instead caused a fall.

### Participants’ experience of the intervention

*Individual interviews* will be conducted 6 months after baseline with participants randomised to the intervention group, to explore and describe their experiences of the intervention, the data collection methods, and the perceived impact on daily life (see online supplemental appendix for interview guide). Digital interviews will be carried out with all participants enrolled in the intervention group. A semi-structured interview guide will be used to enhance trustworthiness. The main areas of inquiry will include experiences of participating in the intervention; the feasibility of the measurements, questionnaires and follow-ups; the impact on daily life; and whether the intervention was perceived as valuable. The interviewer will not be directly involved in delivering the intervention to the informants.

### The RCT

The primary outcome is *self-reported falls during a 6-month period*. A fall is defined as ‘an unexpected event which results in a person coming to rest inadvertently on the ground or floor or other lower level’.^26^ Every other week, the physiotherapist will send a text message to each participant asking whether a fall has occurred or not. Participants who report a fall will receive a phone call from the physiotherapist and be asked to describe the fall event.

Secondary outcomes (questionnaires).

‘Do you think that fear of falling has made you cut down on any activities that you used to do?’ (yes, no, don’t know, prefer not to answer). This is a recommended question for evaluating the effects of fall prevention interventions.^27^

‘How do you rate your ability to avoid falls?’ (visual analogue scale of 0–10).

‘How would you rate your ability to understand what factors increase the risk of you falling?’ (visual analogue scale of 0–10).

‘Has your balance changed since the start of the study? (no, yes—for the better, yes—for the worse).

*Falls Efficacy Scale*^28^ (13 questions measuring whether a person is concerned about falling, rated in four steps from ‘not at all’ to ‘very concerned’).

*Activities-Specific Balance Confidence Scale* (confidence in remaining balance during 16 activities,^29^ rated from 0% for ‘not at all confident’ to 100% for ‘totally confident’). This scale has a satisfactory validity for those with mild-to-moderate MS, and discriminates between multiple fallers and non-fallers.^30^

*12-item MS Walking Scale* (12 statements about limitations of walking ability due to MS in the past 2 weeks,^31^ rated from ‘not at all’ to ‘extremely’). This scale has been translated into Swedish and validated.^32^

### Measures to describe level of functioning

The *Timed Up and Go Test* measures basic mobility in terms of the time taken to rise from a chair, walk 3 metres, turn, walk back and sit down again.^33^ It is valid for people with MS.^34^

The *Six Spot Step Test* measures speed, coordination and balance for people with MS.^35^ For measuring walking impairment, it is superior to both shorter and longer walking tests.^36^ The person walks in a zigzag while kicking bricks, using one foot for each round.

The *Symbol Digit Modalities Test* is a quickly administered screening test for measuring cognition in people with MS, using 10 geometric figures paired with 10 numbers.^37^

The *Frändin-Grimby Physical Activity Scale* is a measure of self-perceived physical activity, answered on a scale running from ‘hardly any physical activity’ to ‘hard or very hard physical activity’.^38^

The *Multiple Sclerosis Impact Scale* measures physical and psychological MS-specific outcomes through 29 questions about how MS affects everyday life, answered on a scale ranging from ‘not at all’ to ‘extremely’.^39^

*Demographic data* will include age, sex, time since diagnosis, MS type, number of falls during the past 3 months, ongoing physiotherapy/rehabilitation, walking device, occupation and living conditions.

*Adverse events* related to the intervention will be documented by the treating physiotherapist.

### Sample size

There is a lack of consensus regarding sample size for pilot studies. The present study will include 35–40 participants, which is in line with recommendations.^40^ This is approximately half of the estimated study sample for the full-scale trial. The sample size was calculated assuming that the control arm will experience 2.2 falls per person per month, using results from a meta-analysis.^5^ With the intention of 30% less falls in the intervention group during a 6-month period compared with the control group, and using a two-sided test with 5% significance level and 80% power and assumed overdispersion of 0.2, the estimated sample size for the comparison of rates using negative binomial regression analysis was 36 participants in each arm. For the qualitative parts of the pilot study, approximately 15–20 participants from the intervention group will be enough to achieve information power.^41^

### Randomization

For the randomised studies, a block randomisation with varied block sizes (2–8) for each participating centre will be generated by one of the authors (RU), who is not involved in the intervention. Allocation will be concealed by using sequential numbered opaque sealed envelopes until the intervention or control condition is assigned.

### Statistical methods

The flow of participants through the study will be examined and illustrated according to the CONSORT statement,^42^ including the proportion of participants lost to follow-up.

Analyses will be performed on an intention-to-treat basis by one of the authors (RU) who will be blinded to allocation. Baseline demographic and clinical characteristics of the study participants will be summarised for the intervention and control group separately. Continuous variables will be summarised using the measures of central tendency and dispersion (mean and SD, median and IQR, minimum and maximum values) as appropriate based on the distribution. Categorical variables will be summarised using frequencies and percentages. Since the randomisation implies that baseline characteristics are expected to be balanced between the intervention groups, we will evaluate whether any substantial imbalance may have occurred by chance.^43^ The distribution of outcome data will be examined.

If dropout is<15%, a negative binomial regression model with an offset term will be used to account for follow-up time. If dropout exceeds 15% but is judged to be non-informative (ie, unrelated to the outcome or intervention), the same approach will be applied. If the event rate is not constant over time, a recurrent event model (eg, Andersen-Gill with robust standard errors) will be used.^44 45^ If dropouts are considered informative, a joint frailty model will be applied.^46^ We may consider Cox regression to compare time to the first fall between intervention arms. Estimates will be presented both unadjusted and adjusted for baseline covariates.^43^ Secondary outcomes will be analysed according to the measurement type and distribution. No evidence suggests that outcomes would systematically differ between physiotherapists or between centres therefore we do not expect systematic variation between physiotherapists or participating centres. However, in a sensitivity analysis, centre will be included as a fixed effect (using five dummy variables) in the negative binomial regression model.

### Qualitative analysis

Qualitative content analysis according to Elo and Kyngäs^22^ will be applied for all digital interviews which will be transcribed verbatim into text files by a professional transcription service. The qualitative content analysis process comprises three main phases—preparation, organising and reporting—aiming to describe participants’ experiences in a conceptual form.

### Ethics and dissemination

The study was approved by the Swedish Ethical Review Authority, Stockholm Dept. 4 medicine (ID: 2025-04486-1, date: 2025–08-13). All participants will provide written informed consent (online supplemental appendix 1). Consent forms will be collected by the respective physiotherapist at each centre for the randomised trials, and by the primary investigator for the qualitative study. All data are managed in accordance with Region Örebro’s procedures for research data and the information management plan for research documents. This means that collected data are stored in a pseudonymised format on a dedicated storage area for research data, the FoU Drive, which is designed to meet requirements for handling research data containing sensitive personal information. The FoU Drive is located in Region Örebro County’s own data centre and is part of a central file server solution with redundancy, backup and logging in accordance with Region Örebro County’s procedures. Data are handled at each site in accordance with applicable procedures. Pseudonymised data are sent by mail to the principal investigator, and a copy is retained by each physiotherapist until it has been confirmed that the data has reached the recipient. Findings will be disseminated through peer-reviewed journals, conference presentations and relevant patient organisations.

### Responsibility of the organising site

The physiotherapists will undergo training led by the primary investigator (YN) and the assisting investigator (AW) in designing and delivering the intervention to assure good quality. This will be undertaken by using previously collected descriptions of nine fall situations in workshops where the analysis will be guided by an ICF manual. The fall situations describe falls occurring both indoors and outdoors, at home and at work and among both men and women. The participating physiotherapists will work in pairs to formulate suitable follow-up questions and potential strategies based on each fall description before moving into whole-group discussions. This process enables the physiotherapists to share experiences and reflections and is intended to support the development of a mutual understanding of how to take a fall history and identify strategies. Physiotherapists at each centre will be encouraged to discuss their experiences with one another for learning purposes throughout the study period.

Further discussions will be held in individual digital meetings between the participating physiotherapists and YN and AW when the first three participants randomised to the intervention group are included. During these meetings, the recruitment process, the measurement procedures, the fall-history assessment and the suggested strategies will be discussed. A summary of the discussions will be sent by e-mail to all participating physiotherapists. Recurrent whole-group meetings will be held digitally and physically two to three times each semester throughout the study period to ensure continued fidelity to the intervention and the research process. In addition, it will be possible to discuss strategies with the primary investigator at any time during the study period. These meetings will also be used to communicate any important protocol modifications. Standardised study-specific documents will be used for the registration of outcomes, the description of fall history, the timing of follow-ups and other data collection procedures. The physiotherapists will also be trained to collect the other data and to become familiar with the flow of the study.

## Supplementary material

10.1136/bmjopen-2025-115430 online supplemental file 1

10.1136/bmjopen-2025-115430 online supplemental file 2

## Data Availability

No data are available.
