# Supplementary material for: Individually designed fall prevention strategies compared to generic strategies for mastering complex fall risk situations in people with multiple sclerosis: study protocol of a randomised controlled trial
Source: BMJ Open. 2026 Jul 15;16(7):e115430. doi: 10.1136/bmjopen-2025-115430 (PMC13374452; doi:10.1136/bmjopen-2025-115430)
Supplement: online supplemental file 1 [file bmjopen-16-7-s001.docx]

Appendix 1

Interview guide

INTRODUCTORY QUESTION: You took part in a study focused on fall risk – can you tell me what it was like to participate in this study?

TALKING ABOUT FALL INCIDENTS AND RECEIVING INDIVIDUAL STRATEGIES

- What experiences do you have of discussing fall incidents with a physiotherapist? (in general, in the study)?
- Can you describe what it was like to receive strategies tailored to you to reduce falls? How have you used it?
- In what way does the information you received as a brochure differ from the individual strategy you were given?

TESTS, FORMS, AND FOLLOW-UPS

- At the beginning you met a physiotherapist – what were your experiences of that visit?
- You reported any falls every other week; what are your experiences of that? (benefit, learning, inconvenience, focus on impairments, etc.)

IMPACT ON DAILY LIFE

- Can you tell me whether participating in the study has had any impact on your everyday life?
- How would you describe your ability to manage fall risk today?

ADDED VALUE OR NOT

- Would you recommend someone else ask for individual strategies to reduce fall risk?

IS THERE ANYTHING ELSE YOU WOULD LIKE TO SHARE?
