# Supplementary material for: Individually designed fall prevention strategies compared to generic strategies for mastering complex fall risk situations in people with multiple sclerosis: study protocol of a randomised controlled trial
Source: BMJ Open. 2026 Jul 15;16(7):e115430. doi: 10.1136/bmjopen-2025-115430 (PMC13374452; doi:10.1136/bmjopen-2025-115430)
Supplement: online supplemental file 2 [file bmjopen-16-7-s002.docx]

# Consent to participate in this research project

I have received oral and/or written information about the study and have had the opportunity to ask questions. I may keep the written information.

I consent to participate in the project *Digitally Supported Intervention for Managing Complex Cases in Multiple Sclerosis: A Multicenter Study*.

• I consent to my results being stored in the manner described in the participant information sheet.

| Place and Date | Signature |
| --- | --- |
|  |  |
|  | Printed name |
|  |  |
