# Supplementary material for: Individually designed fall prevention strategies compared to generic strategies for mastering complex fall risk situations in people with multiple sclerosis: study protocol of a randomised controlled trial
Source: BMJ Open. 2026 Jul 15;16(7):e115430. doi: 10.1136/bmjopen-2025-115430 (PMC13374452; doi:10.1136/bmjopen-2025-115430)
Supplement: Supplementary file 3 [file bmjopen-2025-115430.reviewer_comments.pdf]

PEER REVIEW HISTORY

BMJ Open publishes all reviews undertaken for accepted manuscripts. Reviewers are asked to complete a checklist review form and are provided with free text boxes to elaborate on their assessment. These free text comments are reproduced below.

ARTICLE DETAILS

Title (Provisional)

Individually designed fall prevention strategies compared to generic strategies for mastering complex fall risk situations in people with multiple sclerosis: study protocol of a randomised controlled trial

Authors

Nilsagård, Ylva; Udumyan, Ruzan; Halleberg Nyman, Maria; Wäneskog, Anna

---

VERSION 1 - REVIEW

**Reviewer**	**1**
**Name**	**Zhao, Yinshan**
**Affiliation**	**University of British Columbia**
**Date**	**26-Feb-2026**
**COI**	**None**

This RCT aims to examine personalized fall prevention strategies compared to generic strategies in people with MS. The participants will be randomized 1:1 to the study arms. The sample size of the full study is 72 with an embedded pilot study of 35 – 40 participants. The primary outcome is number of falls during 6 months. Secondary comes are self-reported outcomes related to falling. The pilot study also has a qualitative component to inform the full study. The rationale and methods are clearly presented in the protocol.

It would be helpful to discuss the potential advantages and risks of including an internal pilot study and any strategies to mediate the risks.

How many physiotherapists are expected to participate? Will the variability across physiotherapists be accounted for either in the randomization or in the analysis?

Sample size, I assume that the overdispersion parameter (0.2) is the alpha in the following formular: Var(Y) = mu + alpha * mu^2. Could the author confirm this? Is the degree of overdispersion (0.2) also based on reference 5?

Please clarify lost to follow-up will be handled?

A minor point: Some text is redacted (XXXX).

**Reviewer**	**2**
**Name**	**Kierkegaard, Marie**
**Affiliation**	**Karolinska Institute, Department of Neurobiology, Care Sciences and Society**
**Date**	**14-Apr-2026**
**COI**	**None**

Thank you for letting me review this nice and well-written study protocol. I have only mainly minor remarks, mostly concerning the need for some clarifications as stated below.

Abstract

Page 1

Line 40: “will evaluate whether a fall prevention strategy” – isn’t it rather individual fall prevention strategies, i.e., one single strategy for each person seems not sufficient given the fact that there are probably several different fall risk factors that need to be addressed.

Line 45: “group will have discussions with a physiotherapist regarding” – clarify if it is one or more occasions that the individual will have a discussion/discussions with the physiotherapist.

Page 2

Line 13: “and secondary outcomes will be self-rating scales” – there is no mention about other variables that are collected at baseline (e.g., SDMT, MSIS-29 Tug etc).

Line 29: “This trial was registered with ClinicalTrials.gov (Protocol no: 258079)” – please provide correct protocol number, think it is NCT07378566

Introduction

Page 4

Line 5-10: “Since fatigue is common in people with MS, interventions using remote solutions may save energy, reducing the travel time and effort needed to attend care visits. Remote solutions can thereby increase the accessibility of an intervention.” – I completely agree, but can the authors clarify how this is related to their intervention. Which remote solution do they mean?

Line 20-27: “The present study responds to that criticism by explicitly studying the effect and experiences of individual counselling for fall prevention strategies among people with MS, bridging over the problem of evaluating interventions that comprise several components.” – Interesting thoughts, though I disagree with your conclusion that you will be bridging over the problem described as every individual will most probably use, or at least told to use, several different strategies, i.e., comprising several components.

Methods and Analysis

Page 5

Line 3-5: “The hypothesis is that the intervention group will report 30% less falls during the 6-month period when the intervention is active, compared to the control group.” – interesting, what is the rationale for choosing a 30% difference between groups?

Line 56-59: “Patient involvement was used when planning the study, including discussion of the relevance of the project as a whole and the specific objectives.” – Please provide more information on patient involvement, how were they recruited? How many patients? Etcetera…

Page 6

Line 13-16: “The collaboration was formalized according to the current guidelines of Region XXXXX County, which include reimbursement to patient representatives.” – Why XXXXX, shouldn’t it be the name of the county?

Line 23-26: “Physiotherapists trained in neurological physiotherapy will include participants at 6 centres in XXXXxxxxx.”- How many physiotherapists? How will you secure that they are trained in neurological physiotherapy? Why XXXXxxxxx? Why not provide information on where these centres are?

Line 42: “The exclusion criteria will be: difficulty filling in self-rating scales” – How will this be judged? Is it decided based on physical and/or cognitive function?

Line 55: “An individual fall preventative strategy, based on a fall risk analysis” – this is really a minor remark, but I still think that the intervention for each individual is not a single strategy but rather several different strategies.

Page 7

Line 8-11: “During a visit to the physiotherapist clinic, the individual will be asked to describe recent fall situations.” – How long will this visit be? Is that the only visit to the physiotherapist?

Line 60: “physiotherapist will formulate and document an individual fall prevention strategy” – still think that there will be several strategies, and also interesting that it is

the physiotherapist that decide and formulate the strategies, does not seem so person-centred.

Page 10

Line 29: "*Feasibility of the data collection procedures will consider"* – can the authors clarify how they will decide if the data collection procedures are feasible.

Page 12

Line 40: "Measures to describe level of functioning" -What is the rationale for these measures? I presume that these are for descriptive purposes as the measures are only used at baseline. It could be described clearer why these measures are included.

Page 14

Line 6-8: "For the randomized studies, a block randomization with varied block sizes for each participating centre will be generated by one of the authors (XX)" – Can the authors clarify which block sizes that they intend to use. Please provide initials instead of XX.

Line 29: "....participants will be summarized for the two intervention groups.." – I presume the authors mean the intervention and control groups

Page 15

Line 20-40: Please provide the correct information instead of al the XXXX

Line 58: "The physiotherapists will undergo training in designing and delivering the intervention" – Please provide more detailed information on the training, who will provide this training, what sort of training, and to what extent.

Page 16

Line 8-10: "Recurrent meetings will be held over the study period to ensure continued fidelity to the intervention." – Please provide information on the extent of these recurrent meetings, and who will be involved in these meetings.

Line 13-15: "Standardized documents will be used for data collection." – can the authors clarify what they mean by this, what will these document cover.

Line 39-55: Please provide information author initials instead of XX.

Page 17

Line 3-8: Please provide information author initials instead of XX.

Line 21-25: Please provide information instead of the XXX

Line 46: Provide the correct information on ORCID Ids

SPIRIT Checklist

There are a few items the authors need to check for accuracy; 3a, 14b, 18 (add table 2), 28b, 31 and 32.

VERSION 1 - AUTHOR RESPONSE

Thank you for your valuable and insightful comments. We have carefully considered all of them and addressed each point in the list below.

**Reviewer 1:**<br>This RCT aims to examine personalized fall prevention strategies compared to generic strategies in people with MS. The participants will be randomized 1:1 to the study arms. The sample size of the full study is 72 with an embedded pilot study of 35 – 40 participants. The primary outcome is number of falls during 6 months. Secondary comes are self-reported outcomes related to falling. The pilot study also has a qualitative component to inform the RCT study. The rationale and methods are clearly presented in the protocol.	Thank you.
It would be helpful to discuss the potential advantages and risks of including an internal pilot study and any strategies to mediate the risks.	Thank you for this opportunity to elaborate on the topic. We have inserted the following supported by a reference:<br>The internal pilot will include centres identified for the main trial to ensure a more realistic assessment of feasibility (recruitment, retention, data collection, adherence) and may provide empirical estimates to refine sample size assumptions for the main trial. If no major protocol changes are required, pilot data will be retained for the final analysis, thereby improving overall study efficiency.<br>To minimize risks of bias and operational inconsistency, progression criteria will be pre-specified, any sample size re-estimation will follow predefined methods, and protocol modifications will be kept to a minimum and fully documented, ensuring consistency between the pilot and main phases and preserving study validity.

How many physiotherapists are expected to participate? Will the variability across physiotherapists be accounted for either in the randomization or in the analysis?	We have clarified the number of physiotherapists participating and added a sentence about systematic differences.<br><br>Changes: Physiotherapists (two per center) trained in neurological physiotherapy will include participants at 6 centres in Sweden:<br><br>No evidence suggests that outcomes would systematically differ between physiotherapists or between centres therefore we do not expect systematic variation between physiotherapists or participating centres. However, in a sensitivity analysis, centre will be included as a fixed effect (using five dummy variables) in the negative binomial regression model.
Sample size, I assume that the overdispersion parameter (0.2) is the alpha in the following formular: Var(Y) = mu + alpha * mu^2. Could the author confirm this? Is the degree of overdispersion (0.2) also based on reference 5?	Yes, the overdispersion parameter corresponds to α in the NB2 parameterization, where $Var(Y) = \mu + \alpha\mu^2$. The Stata sample size calculation following Zhu and Lakkis (2014) is based on this variance structure. (1)<br><br>The degree of overdispersion (α = 0.2) was chosen provisionally, and sensitivity analyses were conducted using a range of values (0.2–1.2). As expected, higher levels of overdispersion require larger sample sizes. We anticipate that the pilot study will provide empirical estimates to refine these assumptions for the main trial, thereby reducing the risk of type II error.
Please clarify lost to follow-up will be handled?	Thank you for your question.<br>We have added this information:<br><br>If dropout is <15%, a negative binomial regression model with an offset term will be used to account for follow-up time. If dropout exceeds 15% but is judged to be non-informative (i.e., unrelated to the outcome or intervention), the same approach will be applied if dropout exceeds 15% but is judged to be non-informative. If the event rate is not constant over time, a recurrent event model (e.g. Andersen-Gill with robust standard errors) will be used (2, 3). If dropouts is considered informative, a joint frailty model will be applied (4). We may consider Cox regression to compare time to the first fall between intervention arms. Estimates will be presented both unadjusted and adjusted for baseline covariates (5). Secondary outcomes will be analysed according to the measurement type and distribution. No evidence

	suggests that outcomes would systematically differ between physiotherapists or between centres therefore we do not expect systematic variation between physiotherapists or participating centres. However, in a sensitivity analysis, centre will be included as a fixed effect (using five dummy variables) in the negative binomial regression model.
**Reviewer 2:** Thank you for letting me review this nice and well-written study protocol. I have only mainly minor remarks, mostly concerning the need for some clarifications as stated below.	Thank you for your kind words.
Page 1 Line 40: “will evaluate whether a fall prevention strategy” – isn’t it rather individual fall prevention strategies, i.e., one single strategy for each person seems not sufficient given the fact that there are probably several different fall risk factors that need to be addressed.	Thank you for this comment. After considering your suggestion, we have changed individual fall prevention strategy to strategies
Line 45: “group will have discussions with a physiotherapist regarding” – clarify if it is one or more occasions that the individual will have a discussion/discussions with the physiotherapist.	Thank you for the opportunity to clarify this. Each participant meets with a physiotherapist at one time physically. Further discussions will take place if reporting falls during the follow-up period.<br>Changes: an extensive discussion and in case of falling during follow-up, further discussions on strategies will be held by phone contact.
Page 2 Line 13: “and secondary outcomes will be self-rating scales” – there is no mention about other variables that are collected at baseline (e.g., SDMT, MSIS-29 Tug etc).	Thank you for pointing that out. We have added a sentence about descriptive measures but chose not to describe them further in the abstract.<br><br>Descriptive measures of disease impact will be used.
Page 2 Line 29: “This trial was registered with ClinicalTrials.gov (Protocol no: 258079)” – please provide correct protocol number, think it is NCT07378566	Thank you for this observation. It is correct that the ID is NCT07378566 and we have corresponded this with Dr Jamie Royle on February the 4th. The registration was late partly due to the shutdowns of administration in the US. We have now changed to the correct ID No.<br>NCT07378566 2025-12-08
Page 4 Line 5-10: “Since fatigue is common in people with MS, interventions using remote solutions may save energy, reducing the travel time and effort	Thank you for giving us the opportunity to clarify this. Follow-ups are managed by phone (sms) and in case of experiencing falls, a new discussion about strategies will follow, also by phone. We have that

needed to attend care visits. Remote solutions can thereby increase the accessibility of an intervention." – I completely agree, but can the authors clarify how this is related to their intervention. Which remote solution do they mean?	information further on in the manuscript but will add information also here.<br>Change: Remote solutions, for example using short message service and phone calls as in the present study, can thereby increase the accessibility of an intervention.
Page 4 Line 20-27: "The present study responds to that criticism by explicitly studying the effect and experiences of individual counselling for fall prevention strategies among people with MS, bridging over the problem of evaluating interventions that comprise several components." – Interesting thoughts, though I disagree with your conclusion that you will be bridging over the problem described as every individual will most probably use, or at least told to use, several different strategies, i.e., comprising several components.	We agree that the participants most probably will use several strategies. What we tried to express was that combining different interventions introduces difficulties to conclude what the effective ingredient (or ingredients) were.<br>We changed the wording to make this more explicit.<br><br>Change: Rehabilitation professionals often use combinations of supervised and non-supervised exercise and advice, to achieve the desired effect. There has been some criticism of combining interventions, as these act as a so-called "black box" and make it difficult to distinguish which components are effective and which are not.
Page 5 Line 3-5: "The hypothesis is that the intervention group will report 30% less falls during the 6-month period when the intervention is active, compared to the control group." – interesting, what is the rationale for choosing a 30% difference between groups?	The choice of using 30% was based on clinical reasoning considering several factors. We have added text and references to clarify this.<br><br>Changes: This was based on clinical reasoning, considering that healthy people also fall (6), that people with MS often falls during everyday activities that are difficult to avoid (7, 8), that behavioural change takes time (9), and, that the control group receives fall-related information that may influence fall outcomes.
Page 5 Line 56-59: "Patient involvement was used when planning the study, including discussion of the relevance of the project as a whole and the specific objectives." – Please provide more information on patient involvement, how were they recruited? How many patients? Etcetera…	We have made the following changes to the text:<br><br>A patient representative with personal experience of falling, recruited by a MS team nurse in Region Örebro County, contributed to the study planning. Patient involvement included discussions about the overall relevance of the project and its specific objectives.

Page 6 Line 13-16: “The collaboration was formalized according to the current guidelines of Region XXXXX County, which include reimbursement to patient representatives.” – Why XXXXX, shouldn’t it be the name of the county?	We sent both a blinded and an unblinded version to the BMJ Open. The resubmitted version is unblinded.<br><br>Change: Region Örebro County
Page 6 Line 23-26: “Physiotherapists trained in neurological physiotherapy will include participants at 6 centres in XXXXxxxxx.”- How many physiotherapists? How will you secure that they are trained in neurological physiotherapy? Why XXXXxxxxx? Why not provide information on where these centres are?	Physiotherapists trained in neurological physiotherapy will include participants at 6 centres in Sweden: Department of Neurology and Rehabilitation Medicine, University Hospital, Örebro; Department of Physiotherapy, Hudiksvall Hospital, Hudiksvall; NeuroRehab Unit, Mälar Hospital, Eskilstuna; Department of Physiotherapy, Gävle Hospital, Gävle; Neurological Rehabilitation Unit, Västerås Hospital, Västerås; and NeuroRehab, Nyköping Hospital, Nyköping.
Line 42: “The exclusion criteria will be: difficulty filling in self-rating scales” – How will this be judged? Is it decided based on physical and/or cognitive function?	Thank you for this question. It will be the respective physiotherapist that uses clinical experience and judgement that checks all inclusion and exclusion criteria. We have clarified this.<br><br>Change: The exclusion criteria are judged by the respective physiotherapist and will be: any difficulty filling in self-rating scales
Page 6 Line 55: “An individual fall preventative strategy, based on a fall risk analysis” – this is really a minor remark, but I still think that the intervention for each individual is not a single strategy but rather several different strategies.	We agree.<br><br>Change: Individual fall preventative strategies
Page 7 Line 8-11: “During a visit to the physiotherapist clinic, the individual will be asked to describe recent fall situations.” – How long will this visit be? Is that the only visit to the physiotherapist?	Yes, exactly, one physical visit. We have added the estimated time.<br><br>Change: We estimate the time for the visit to approximately 90 minutes and will follow-up this estimation during the internal pilot study.
Line 60: “physiotherapist will formulate and document an individual fall prevention strategy” – still think that there will be several strategies, and also interesting that it is the physiotherapist that decide	Thank you for this question. This allows us to refine our writing in this part.<br>Changes: Using the time when the participant is filling in the self-report questionnaires, the physiotherapist will document the agreed individual fall prevention strategies and will then go through it with the participant at the end of the visit. A teach-back method is used, in

and formulate the strategies, does not seem so person-centred.	which participants repeat the strategies back to the physiotherapist to ensure mutual agreement and to reinforce the strategies. Participants are encouraged to share at least one strategy with a next-of-kin after the visit and to place the written strategies somewhere at home where they will be seen daily.
Page 10 Line 29: "Feasibility of the data collection procedures will consider" – can the authors clarify how they will decide if the data collection procedures are feasible.	The PTs will assess if the estimated time for the visit (90 minutes) was sufficient. Furthermore, we will rely on the PTs' clinical judgement whether the effort to fill in the questionnaires and perform the physical and cognitive tests seems reasonable or not. Finally, we will include questions on this topic in the interviews.<br><br>1) Changes: if the estimated time for the visit (90 minutes) was sufficient<br>2) the physiotherapists' clinical judgement of participant effort to fill in the questionnaires and perform the physical and cognitive tests, discussed repeatedly during the study period.
Page 12 Line 40: "Measures to describe level of functioning" - What is the rationale for these measures? I presume that these are for descriptive purposes as the measures are only used at baseline. It could be described clearer why these measures are included.	The rational for the measures are exactly what the subheading describes. "Measures to describe level of functioning". We believe that it is important when interpreting the results to know the level of functioning of the study sampling. We have considered how to write this clearer but fall back on the sub-heading. We are open for suggestions.
Page 14 Line 6-8: "For the randomized studies, a block randomization with varied block sizes for each participating centre will be generated by one of the authors (XX)" – Can the authors clarify which block sizes that they intend to use. Please provide initials instead of XX.	Change: (RU)<br><br><br>Yes of course, thank you for pointing this out.<br>Change: (2-to 8)
Line 29: "....participants will be summarized for the two intervention groups.." – I presume the authors mean the intervention and control groups	Yes, thank you. The control group does get an intervention (fall information) but in line with the reporting in this manuscript it would be much clearer to stick with intervention and control group.<br>Change: for the intervention and control group separately

Page 15 Line 20-40: Please provide the correct information instead of al the XXXX	The study was approved by the Swedish Ethical Review Authority, Stockholm Dept. 4.<br>All data are managed in accordance with Region Örebro's procedures for research data<br>The FoU Drive is located in Region Örebro County's own data center<br>Region Örebro County's procedures.
Line 58: "The physiotherapists will undergo training in designing and delivering the intervention" – Please provide more detailed information on the training, who will provide this training, what sort of training, and to what extent.<br>Page 16 Line 8-10: "Recurrent meetings will be held over the study period to ensure continued fidelity to the intervention." – Please provide information on the extent of these recurrent meetings, and who will be involved in these meetings.	Thank you for this comment. We are happy to elaborate on this.<br>Changes:<br>The physiotherapists will undergo training led by the primary investigator (YN) and the assisting investigator (AW) in designing and delivering the intervention to assure good quality. This will be undertaken by using previously collected descriptions of nine fall situations in workshops where the analysis will be guided by an ICF manual.<br><br>The fall situations describe falls occurring both indoors and outdoors, at home and at work, and among both men and women. The participating physiotherapists will work in pairs to formulate suitable follow-up questions and potential strategies based on each fall description before moving into whole-group discussions. This process enables the physiotherapists to share experiences and reflections and is intended to support the development of a mutual understanding of how to take a fall history and identify strategies. Physiotherapists at each centre will be encouraged to discuss their experiences with one another for learning purposes throughout the study period.<br><br>Further discussions will be held in individual digital meetings between the participating physiotherapists and YN and AW at the inclusion of the first three participants randomized to the intervention group. During these meetings, discussions will be held about the recruitment process, the measurement process, the taking of fall history and the suggested strategies. A summary of the discussions will be sent by e-mail to all participating physiotherapists. Recurrent whole-group meetings will be held digitally and physically over the study period two to three times each semester to ensure continued fidelity to the intervention and the research process. In addition, it will be possible to discuss at any time with the primary investigator during the study period.

Line 13-15: “Standardized documents will be used for data collection.” – can the authors clarify what they mean by this, what will these document cover.	Yes of course.<br>Change: Standardized study-specific documents will be used for registration of outcomes, description of fall history, time for follow-ups, etc.
Line 39-55: Please provide information author initials instead of XX. Page 17 Line 3-8: Please provide information author initials instead of XX. Line 21-25: Please provide information instead of the XXX	Yes, this is now provided.
Line 46: Provide the correct information on ORCID Ids	Yes, in this unblinded manuscript, the ORCID IDs are shown:<br><br>Ylva Nilsagård ORCID 0000-0002-9760-3785<br>Ruzan Udumyan ORCID 0000-0001-9204-1165<br>Maria Hälleberg Nyman ORCID 0000-0003-0460-3864<br>Anna Wäneskog ORCID 0000-0002-2598-2317
SPIRIT Checklist There are a few items the authors need to check for accuracy; 3a, 14b, 18 (add table 2), 28b, 31 and 32.	**3a** the names and affiliations are provided<br><br>The roles of protocol contributors are provided<br><br>**14b** is reported under Setting<br><br>**18** – OK<br>**31** we added a sentence to clarify this more:<br>Change: These meetings will be used to communicate possible important modifications.<br>**32**- we added a sentence<br>Changes: Consent forms will be collected by the respective physiotherapist at each center for the randomized trials and by the primary investigator for the qualitative study.

VERSION 2 - REVIEW

**Reviewer**	**1**
**Name**	**Zhao, Yinshan**
**Affiliation**	**University of British Columbia**
**Date**	**10-Jun-2026**
**COI**	

I have no additional comments.

Reviewer **2**

Name **Kierkegaard, Marie**

Affiliation **Karolinska Institute, Department of Neurobiology, Care Sciences and Society**

Date **01-Jun-2026**

COI

Thank you for a nicely revised manuscript. I have only two very minor remarks.

1) The first sentence in the abstract seems incomplete. Perhaps it should read: Of individuals with mild-to-moderate multiple sclerosis (MS), 56% report falling at least once during a 3-month period.

2) In strengths and limitations you state: "Patient representatives have been partners throughout the designing and planning of the study", but you only involved one person so it should read: A patient representative has been a partner throughout the designing and planning of the study.

VERSION 2 - AUTHOR RESPONSE

Reviewer: 2
Dr. Marie Kierkegaard, Karolinska Institute, Region Stockholm
Comments to the Author:
Thank you for a nicely revised manuscript. I have only two very minor remarks.
1) The first sentence in the abstract seems incomplete. Perhaps it should read: Of individuals with mild-to-moderate multiple sclerosis (MS), 56% report falling at least once during a 3-month period.
2) In strengths and limitations you state: "Patient representatives have been partners throughout the designing and planning of the study", but you only involved one person so it should read: A patient representative has been a partner throughout the designing and planning of the study.

Reviewer 2:

1) I have added “Of” in the beginning of the first sentence in the abstract.
2) I have changed the sentence according to the reviewer’s suggestion: “A patient representative has been a partner throughout the deigning and planning of the study”.
